# Buffalo Medical Club

**Published:** 1877-06

**Authors:** E. N. Brush


ART. III.—*Buffalo Medical Club.*—E. N. Brush, M. D.

At the regular meeting held at the residence of Dr. Mynter, on Wednesday evening, April 18th, Dr. S. G. Dorr read a paper upon "Improvements in operations upon the cranial bones and conditions warranting the same," in which he argued in favor of two propositions, and described and exhibited a mechanical appliance for facilitating the removal or elevation of depressed portions of the skull, which he considered superior to the trephine, or any other means hitherto in use, especially for the smaller fractures.

The two propositions were: First, that all tumors of the scalp resulting from violence and presumably containing blood, pus or serum, should be at once incised; and second, that in all fractures of the skull with recognizable depression, operative interference is required as well in simple as in compound fractures, without waiting for symptoms of compression or irritation of the brain; and further, that when there are symptoms of brain irritation without apparent depression, such interference is demanded, provided the operation can be so conducted as not to be more grave than the injury which it is intended to palliate. And he thought that the mechanical means referred to above would enable us to fulfil this provision.

In reference to the first proposition, Dr. DORR said that there is no difference of opinion about incising when the tumor contains pus, but there is great difference about the course to be pursued when the tumor contains blood. Such tumors may form beneath the occipito frontalis muscle or its aponcurosis, when they would be diffuse and fluctuating, or beneath the the pericranium, when they would be firm and unmovable. If the skull is fractured and the pericranium unruptured, the blood taking the direction of least resistance, may disseier up the dura mater and the tumor may become wholly or in part intra-cranial. Dr. DORR thinks all such tumors should be at once incised, believing that it can do no harm, that it may avert the consequences of suppuration degeneration, and that it may enable us to discover a fracture which otherwise might escape observation, deeming such knowledge of greater importance than any possible danger which might arise from thus converting a simple into a compound fracture. He quotes authorities both for and against his proposition, and presents the following statistics from the report of Surgeon General Barnes of scalp injuries during the war of the rebellion. There were:

| | Cases. | Deaths. |
|---|---|---|
| Incised wounds of the scalp................ | 282. | 2.1 per cent. |
| Gunshot wounds of the scalp.............. | 7739. | 1.1 " " |
| Contussions and larcerations of the scalp... | 331. | None. |

He thinks this good evidence that scalp injuries are not in themselves very dangerous, and that the mortality from incised and gunshot wounds may be largely due to undiscovered fractures, particularly of the inner table.

In support of the second proposition, Dr. Dorr commented upon and illustrated by diagrams the well known fact that in depressed fracture of the skull the inner table is usually more extensively broken than the outer one, so that the edges of the inner plate are generally over-lapped by those of the outer table. He doubted whether the internal table was ever fractured except over a sinus without a corresponding fracture of the internal table; while on the other hand the inner table may be splintered while the outer is unbroken. These purely internal fractures are the most dangerous. Thus, during the war twenty cases of fracture of the internal table alone are reported, and of this number nineteen died. Dr. Dorr could not understand how a diagnosis of fracture of the internal table alone could be verified during life, but he wished to state his belief in the plainest terms that where there is any recognizable depression of the external table in fractures of small extent, such cases are of the nature of internal fracture.

While the essayist admitted that it was best not to interfere locally with a fracture without depression so long as there were no symptoms of brain irritation, he presented the following figures from the Surgeon General's report. There were during the war, 2,911 fractures of both tables without known depression, of which number 64.6 per cent. died; also there were 364 cases of depressed gunshot fracture, and only 35.4 per cent. died. This result could only be accounted for in the writer's opinion, either by the neglect of surgical interference in the first class of cases, or by the effects of concussion. It is claimed by some writers that where the skull is broken and depressed, the blow is thereby deprived of a portion of its force, and what would have been concussion, if the skull had not broken in, is thereby converted into depression. Dr. Dorr also quoted other figures from the same source with a view to show that less operative interference had not increased the danger from fracture of the skull, but rather that the mortality had been less when there had been such interference than when there had not.

The symptoms of compression immediately following fracture are due to mechanical causes and may be immediately relieved by elevation of the bone, but the coma or delirium following more remotely after simple fracture are connected more or less with inflammatory action.

In reference to mecanical improvements for operating in these cases, Dr. DORR said that the difficulty in elevating a depressed fragment into its original position, or in removing a fragment through the external opening arose largely from the fact of the inner table being more extensively fractured than the outer one, and being overlapped by the latter. The trephine failed to obviate this difficulty and in small fractures removed too much bone, and that only at one point. The Hays saw is somewhat effective for cutting in straight lines, but as the smaller fractures are more or less curved, sometimes forming irregular circles, this instrument is of restricted use. An instrument which would readily remove the projecting outer table all around the fracture would greatly simplify and render less dangerous, either elevation or removal.

"I here show you a saucer shaped saw, or burr, with which circular cuts can be made. If the saw is more curved, the cut made with it will form a part of a smaller circle. If it is more straight, also will the cut be more straight. With this instrument the the outer table round the margin of a fracture can be cut away, and the depressed splinters loosened from their attachments to the sound bone. Small trephines or drills made of various shapes and sizes are found to be very effective. The emory or corundum wheel, I think, presents qualities which should recommend it as a useful instrument in removing bone. It will not cut flesh, with it there would be no danger of injury to any intra-cranial substance. Its fault is that it becomes rapidly fowled or glazed with blood and fat, and would require frequent cleaning or changing. In operations on the skull I think it very effective if properly made. I have been able to remove by it patches of bone fron a child's skull with facility and safety from injury to other structures. The mode of driving these instruments is the same in all. I have borrowed and brought here to-night a dental engine, it being the best motive power at my command for illustrating practically the operation of the different instruments I have shown you."

The dental engine referred to is used by dentists in some of their operations upon the teeth. The mode of obtaining the power is immaterial. Its peculiarity consists in transmitting the power by means of a flexible wire shaft, running through a flexible tube. The extremity of this tube may be held in the hand, and the little circular saw or other instrument being fixed to the end of the shaft, can be caused to rotate with rapidity and force, and can be manipulated at will.

The saw and drills penetrate bone with facility.

In the discussion of the above paper, Dr. MYNTER said that many attempts had been made to produce a circular saw fitted for surgical use but without much success. He thought the motive power suggested by Dr. DORR supplied the deficiency and might be used for many purposes, as for instance, in sequestrotomy.

